# Saccade reaction test for the assessment of cognitive readiness

**DOI:** 10.3389/fneur.2026.1739645

**Published:** 2026-02-03

**Authors:** Jun Maruta, Jamshid Ghajar

**Affiliations:** 1 Department of Neurology, Icahn School of Medicine at Mount Sinai, New York, NY, United States; 2 Department of Rehabilitation and Human Performance, Icahn School of Medicine at Mount Sinai, New York, NY, United States; 3 Brain Trauma Foundation, Palo Alto, CA, United States

**Keywords:** attentional signatures, brain health, cognitive profiling, eye movement, foveal vision, oculomotor, performance variability, video-oculography

## Abstract

**Background:**

Cognitive performance such as rapidly reacting to a target or making correct decisions can directly impact task effectiveness in military, emergency, or athletic settings. Saccades are rapid changes in gaze that support recognition and analysis of objects of potential interest in human vision whose acuity rapidly degrades away from the center. The saccade behavior is highly selective and controlled and thus is an expression of attention. We implemented a two-dimensional reactive saccade task to quantify attention performance.

**Methods:**

We studied a sample of 169 healthy individuals aged 8–82 years old (39% male), 37 of whom were retested 1–3 months later. Subjects viewed a target presented in a randomized spatiotemporal sequence, and associated timings of saccade initiation and gaze arrival were registered. Individuals’ performance was characterized with the mean and standard deviation of the reciprocals of these measures (1/time, postulated to represent the cortical decision speed).

**Results:**

The intraclass correlation between the test and retest measures varied from 0.60 to 0.74. The reaction speed showed a tendency to become faster and less variable during development in childhood through young adulthood and thereafter become slower and more variable, with best performance tending to be seen in the 20s.

**Conclusion:**

We verified inter-individual variability, within-individual stability, and across-age differences in the performance on a reactive saccade task. A quick assessment of attentional traits or states with saccade reaction metrics, aided by rapidly developing technology, may provide utility in a cognitive readiness test that can inform task assignment or return-to-duty/play decisions.

## Introduction

Cognitive performance such as rapidly reacting to a target or making correct decisions can directly impact task effectiveness of military or emergency operations, or athletic execution. A long list of factors that have been identified to affect cognitive performance includes insufficient sleep, stress, mental fatigue, intoxication, diet, aerobic fitness, and age (1–5). In military settings, the US Department of Defense in particular has maintained long-standing interest in monitoring the cognitive readiness of service members with efforts including the development of batteries of reaction time- and accuracy-based tests of a variety of cognitive domains, such as Automated Neuropsychological Assessment Metrics (ANAM) and Defense Automated Neurobehavioral Assessment (DANA) (6–9). In sports, a history of concussion, insufficient sleep, and attention deficit hyperactivity disorder (ADHD) can increase the risk of injury (10–13). Further to the point, neurocognitive assessment with tools such as the Immediate Post-Concussion Assessment and Cognitive Testing (ImPACT) and ANAM is an essential component of return-to-play decisions after head or orthopedic injury (14–16). Despite such efforts, the basis of cognitive readiness is incompletely understood (5, 9, 17, 18).

Acuity in human vision rapidly degrades as the image projected on the retina is moved from the foveola, the central area that extends only about 1.25° in visual angle (19, 20). Consequently, the gaze, defined as the orientation of this small region in the visual space, must be directly aligned with objects of potential interest for recognition and analysis. In addition, the relative sluggishness of the initial integration at the photoreceptor level in the retina is such that light flickers at as low as 50 Hz is detected as continuous, and retinal slip as low as 1°/s seriously degrades visual acuity, which thus calls for support by gaze holding mechanisms including fixation eye movements, the vestibulo-ocular reflex, and smooth pursuit (21, 22). Necessarily, to scan and reconstruct the visual environment, humans make several saccades every second while awake (20, 22–24). While fixation eye movements that take place between saccades support high visual acuity, saccades rapidly shift the gaze, with peak velocities that can reach several hundred degrees per second in a stereotyped relationship to the amplitude of excursion (21, 22, 25, 26). This highly selective and controlled behavior is by definition an expression of attention. Indeed, the neural networks for attentional and oculomotor controls demonstrated by functional magnetic resonance imaging (MRI) closely overlap, supporting that attention is overtly expressed by the gaze (27–29). Perhaps counterintuitively, however, the neural difference between overt and covert attention is primarily due to the contribution of an additional, competing neural computational process required for suppression of eye movement in covert orientation of attention (29–32).

Saccade generation is cognitive activity foundational to interacting with the environment, i.e., readiness. Reacting to an externally triggered visual event by directing the gaze to the source location is a critical component of saccadic behavior that takes years to develop and then gradually diminishes with aging (33–35). Saccade reaction time is thought to reflect cerebral cortical decision time, show individual differences, and as with other cognitive abilities, be affected by different physiological, metabolic, and neurological conditions (22, 30, 31, 36, 37). For example, saccade reaction times have been reported to increase in amateur boxers after a bout, followed by a recovery over several days (38). Saccade reaction is slower and more variable in children with ADHD compared to neurotypical peers (39). Further, saccade reaction is sped up after dosing with methadone in opioid addicted individuals (40). Since eye movement can be recorded quickly, objectively, and non-invasively with video-oculography, quantifying the performance on a standardized task allows measurement of attention functioning, providing an alternative or supplement to traditional neurocognitive approaches that often also rely on reaction time measurements. We thus implemented a two-dimensional reactive saccade task to aide such quantification.

## Methods

### Sample

We studied a cross-sectional sample of 169 healthy individuals aged 8–82 years old (39% male individuals), 37 of whom further provided retest data 1–3 months later. The study protocol was reviewed and approved by the Institutional Review Board of Weill Cornell Medical College. Prior to data collection, written informed consent by adult subjects, or legal guardians of minor subjects with the minors’ assent, was obtained in accordance with the Declaration of Helsinki 2013 (41). Subjects were recruited via flyers posted at various facilities including colleges, office buildings, hospitals, and community centers in and around the New York City area. Participation required a minimum age of 7 years, a high school diploma or equivalent for those over the age of 18 years, and normal (or corrected to normal) vision. Potential subjects were screened for eligibility through interviews conducted over telephone. Adult eligibility was based on the individual’s responses to screening questions, and pediatric eligibility on a legal guardian’s responses to these same questions. Individuals were excluded for a prior history of traumatic brain injury (including concussion with loss of consciousness), substance abuse, a known neurologic disorder, or a known psychiatric condition (including ADHD and sleep disorder). Family history of psychiatric disorders was not obtained.

A total of 187 (39% male) subjects were initially enrolled, but one discontinued participation. Visually guided saccade reaction performance (described below) was tested in the remaining 186 subjects. The study design called for recruitment of up to one third of the enrolled subjects to also undergo MRI as well as be retested between 1 and 3 months after the initial testing. A report has been published previously on dynamic visual tracking and simple visuo-manual reaction performance from this cohort, not overlapping with the present analysis (42). MRI results of the adult portion of the study cohort have also been published (43). The saccade task eye movement records of 17 individuals were deemed invalid for excessive blinks (see *Eye movement data processing*, below) (nine incidents), recording failure (five incidents), or calibration failure (three incidents). Forty-four of the 186 subjects were tested again, of whom 37 produced valid recordings on both sessions.

### Saccade task

**Figure 1:**
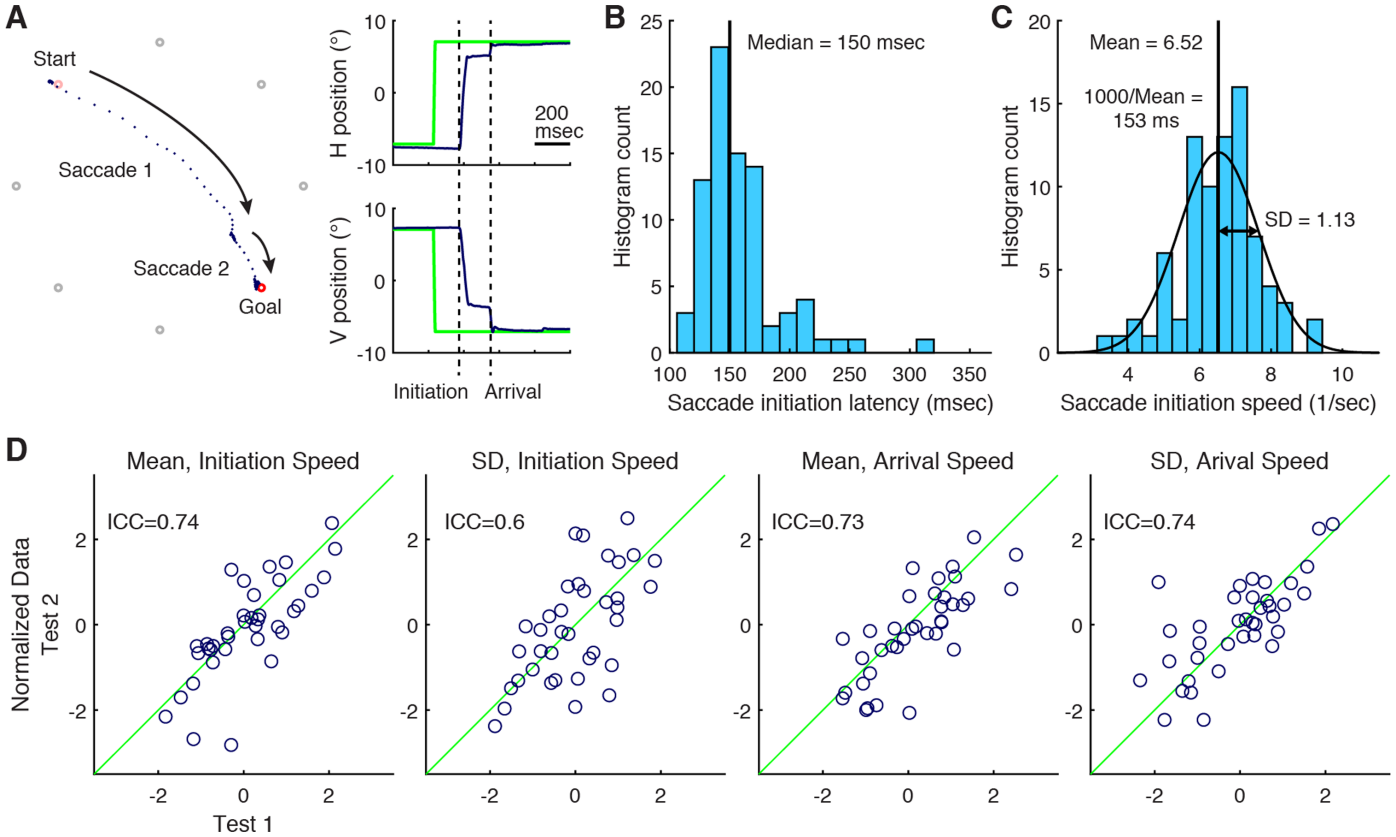
Characterization of saccadic reaction and test–retest reliability. **(A)** An example of eye movement reaction to a sudden change in the target location. Two saccades were executed to reach the target after it moved by 20° in visual angle from the top-left to the bottom-right location, indicated with pink and red outlines, respectively. Other locations where the target might appear are shown in gray. Each dark blue dot indicates eye position sampled at 500 Hz. The insets show plots of the target (green) and gaze (dark blue) locations in time in the horizontal and vertical dimensions. **(B)** A histogram representation of the distribution of saccade initiation times of a single subject over 82 measurements. **(C)** A histogram representation of the distribution of the reciprocals of saccade initiation times of the same subject. **(D)** Test–retest correlograms of saccade performance metrics after their distributions among 37 subjects were normalized with Box-Cox transformation and then standardized. The green diagonal lines indicate lines of equality.

Subjects performed the saccade task implemented using the Experiment Builder software on a video-based eye tracker integrated with stimulus presentation (EyeLink 1000, SR Research Ltd., Mississauga, ON, Canada) in a setup that was described previously (42, 44). Briefly, the subject was seated in a normally lit room with the head stabilized using a head- and chinrest during testing while viewing an LCD computer screen, placed 47.5 cm from the eyes and subtending 53° (horizontal) by 35° (vertical) in visual angles (SyncMaster 2233RZ, Samsung, Seoul, South Korea). Prior to testing, each subject’s vision was screened using a hand-held eye chart to verify normal or corrected-to-normal vision, defined as an acuity of 20/30 or better. Eye position was calibrated by having the subject fixate on a visual target of 0.5° in visual angle, with a 0.2° black dot in its center, that were presented against a black background at the center of the screen and at eight evenly spaced locations on a circle of a 10° radius (left, right, up, down, and intermediate locations) (Figure 1A). The eye tracker recorded the gaze positions in terms of coordinates mapped on the computer screen with which the target was shown. Eye movement was recorded binocularly at 500 Hz.

Reactive saccade performance was assessed using a task that mirrored the calibration procedure. The subject was instructed to focus on the target as it appeared at different locations. During a trial, a central fixation target was first presented, and then the target moved 40 times randomly among the eight peripheral locations, each positioned 10° in visual angle from the center. There was no time gap between the disappearance and reappearance of the target from one location to another beyond that introduced by the LCD screen refreshed at 120 Hz. The trial was concluded with the presentation of a central fixation target, but presumably the subject did not anticipate this event. Accordingly, there were 41 unpredictable changes in the target location in a single trial. Two trials were administered consecutively.

The timing of the target jump was also made unpredictable. The eye tracker automatically identified the arrival of the gaze of either eye at the target in real time when the computed gaze location fell within a 3° square window with the target at its center and stayed there for a minimum of 200 ms. When this event was detected, the target stayed in place for an additional duration of 800–1,500 ms chosen from a uniform random distribution, and then moved to the next location. If the eye tracker failed to detect a gaze fixation on the target, the target stayed in place for a maximum of 2,000 ms. The total testing time per subject, including eye position calibration and task instructions, was about 4 min.

### Eye movement data processing

Eye movement data were analyzed with automated algorithms using a custom MATLAB program (The MathWorks, Natick, MA, USA). Given the small angle of oculomotor range required for the task (±10°), horizontal and vertical eye and target positions were expressed in visual angle by a simple linear conversion from those expressed in terms of the coordinates of the display monitor.

Eye position time series were 2-point differentiated to obtain eye velocity. The horizontal and vertical eye velocity series were smoothed with a 5-point moving-average filter and combined in a Pythagorean manner to represent the instantaneous speed. A saccade was detected when the eye speed exceeded a 30°/s threshold while still accelerating. Once such an event was identified and marked as saccade initiation, the search for a next saccade was resumed from the point in the data series corresponding to passage of at least 50 ms in time and the return of the eye speed below 30°/s. These criteria prevented falsely recognizing miniature eye movement during fixation as a saccade made in reaction to a target move or a portion of an ongoing large saccade as initiation of a new saccade (22, 25, 26, 45, 46). The search for saccades continued until the end of the record was reached.

Intervals during which the pupil was occluded or unidentified due to blinks and other events were padded with ±100 ms periods and excluded from further analysis. In addition, demi-blinks were identified as rapid, incomplete shrinking in the pupil size, and were also excluded from analysis.

A valid response to a step change of target position was defined by the following criteria: (1) the eye was open when the target moved; (2) the gaze was initially at least 3° away from the new target position; (3) the gaze arrived within 3° from the center of the target within 1,500 ms; and (4) the gaze stayed in the latter area for a minimum of 100 ms. For each valid run, the first time point that satisfied the third condition, if there was no blink in the intervening time, was recorded as the arrival time. A correct response was considered to have been achieved by a single or multiple saccades that incrementally reduced the gaze distance from the new target location, instances of which included corrective saccades following an over/undershooting relative to the target position (Figure 1A). Further, in order for a saccade to be considered to be a reaction to a target move, the latency of the saccade initiation needed to be larger than 75 ms, which thus excluded what are known as express saccades (47). The initiation time of the first reactive saccade in each valid run was recorded as the initiation time, if there was no blink between the target move and the saccade. The criteria were applied to the recording of each eye separately, and the shorter of initiation or arrival latency between the two eyes was, respectively, registered as the initiation or arrival latency associated with each target move. Invalid responses were excluded from further analysis. The accuracy of the automated outputs was inspected through an interactive visual interface that allowed manual corrections, if appropriate. A valid test consisted of >95% of runs with the gaze starting away from the target and landing near the target (meeting Criteria 2, 3, and 4, i.e., minimum evidence for task engagement or sufficient recording quality) and >85% registration of valid arrival time (having no blinks between the target move and the gaze arrival).

### Outcome measures

Within an individual, the saccade reaction times are known to be variable and be distributed with a positive skew (Figure 1B). It is postulated that this variability is a consequence of the variable rate, or speed, of the neural decision for saccade initiation; correspondingly, the reciprocals of reaction times (1/time) are nearly normally distributed (Figure 1C) (23). Therefore, individuals’ saccade reaction was characterized with the mean and standard deviation of the reciprocals of initiation latencies, i.e., initiation speeds. Under the stipulation of the near symmetry of the distribution of speeds, their mean and median should be approximately equal, and consequently, the reciprocal of the mean speed should approximate the median latency (Figures 1B,C).

An accurate initial saccade should result in an arrival time delayed only by the saccade duration, which would be just tens of milliseconds and determined essentially by the saccade amplitude alone (25). However, multiple saccades and intervening fixations were often made to ultimately bring the gaze near the target, which variably lengthened the arrival time (Figure 1A). Thus, short and long arrival times were considered, in a broad sense, to, respectively, represent the accuracy of the initial saccade and inefficient execution of attentional shifts. Since the skewed distribution of arrival times made their mean and standard deviation unreliable measures, and to be consistent with the characterization of saccade initiation, the mean and standard deviation of the reciprocals of arrival times, i.e., arrival speeds, were used.

### Statistical analysis

The intraclass correlation coefficient (ICC) with one-way random effect model was computed to determine the level of test–retest agreement (48). ICC ranges from 0 to 1, with the latter value indicating a perfect match. Since the computation of ICC assumes normality of the data and is biased by the skewness of the data, the raw data were transformed with a Box–Cox transformation. The parameter of the transformation was chosen so that the absolute value of the skewness of the distribution of the transformed data was minimized.

A paired *t*-test was used to reject or accept the null hypothesis that no systematic difference existed between test–retest measurements for the select individuals who were tested twice. The alpha level was set at *p* = 0.05. Holm-Bonferroni method (49), a sequentially rejective Bonferroni test, was used for multiple comparisons. The effect size of the difference between the means of the two measurements was examined with Cohen’s d, defined as the mean difference divided by the corrected sample standard deviation (50).

The U-shaped trend of age-dependent performance improvement and decline was described with a quadratic regression model. The independent variable of the model was a natural logarithm transformation of age in years plus one, so that a deceleration of changes with age could be accounted for and the transformed values would always be positive (42). The validity of the fit was tested against a constant model using an F-test. Further, the residuals of the fit were separated by sex, and a two-sample t-test was applied to examine a possible sex difference in the performance measures.

## Results

### Performance repeatability

The intraclass correlation between the test and retest measures varied from 0.60 to 0.74 (Figure 1D). Thus, the task showed overall good individual performance repeatability established relative to the inter-individual variability. However, we detected a statistically significant systematic difference between the test and retest outcomes of mean arrival speed, suggesting slower arrival for second testing, albeit with a small effect size (|t (36)| = 2.68, *p* = 0.011, d = 0.44).

### Reaction speeds vs. age

**Figure 2:**
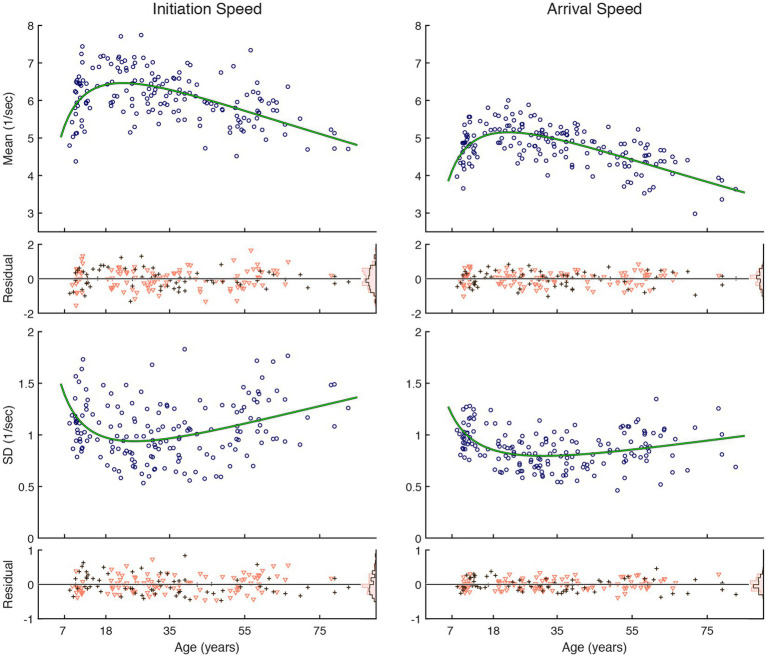
Reactive saccade performance as a function of age. Circular markers indicate individual scores. Green lines indicate regression model fits. See Table 1 for model summary statistics. The residuals, shown separately for female (∇) and male (+) subjects, scatter around zero across age, and their distributions are summarized as histograms in insets on the right edge shown using corresponding color schemes. See Table 2 for summary statistics examining possible sex differences.

Inter-individual variability was large across ages, but the reaction speed, both for saccade initiation and gaze arrival, showed a tendency to become faster and less variable during development in childhood through young adulthood and thereafter become slower and more variable (Figure 2). Changes were faster during development than aging, and the trajectory that followed a U-shaped trend could be approximated by a quadratic function of log-transformed age. The fit was statistically significantly better than a constant model (Table 1). The trend curve of saccade initiation speeds peaked with 6.5/s at the age of 22.5 years old, corresponding to a median latency of 154 ms, while that for initiation speed variability was smallest at the age of 25.7 years old. The trend curve of gaze arrival speeds peaked with 5.2/s at the age of 22.0 years old, corresponding to a median latency of 194 ms, while that for arrival speed variability was smallest at the age of 31.4 years old. A sex difference in the performance measures was not detected (Table 2).

**Table 1:** Summary quadratic regression model statistics of visual tracking performance relative to a function (*X*) of age.

Mean, initiation speed					Mean, arrival speed				
Term	B	SE	*t*	*p*	Term	B	SE	*t*	*p*
*X* ^2^	−0.983	0.135	−7.258	0.000	*X* ^2^	−0.921	0.096	−9.575	0.000
*X*	6.205	0.900	6.897	0.000	*X*	5.778	0.640	9.031	0.000
Constant	−3.332	1.460	−2.282	0.024	Constant	−3.902	1.039	−3.757	0.000
*R*-squared value: 0.293					*R*-squared value: 0.438				
Adjusted *R*-squared value: 0.285					Adjusted *R*-squared value: 0.431				
*F*-statistics vs. constant model: 34.5, *p* < 0.001					*F*-statistics vs. constant model: 64.7, *p* < 0.001				

SD, initiation speed					SD, arrival speed				
Term	B	SE	*t*	*p*	Term	B	SE	*t*	*p*
*X* ^2^	0.310	0.063	4.965	0.000	*X* ^2^	0.203	0.039	5.242	0.000
*X*	−2.039	0.416	−4.907	0.000	*X*	−1.413	0.258	−5.486	0.000
Constant	4.287	0.675	6.355	0.000	Constant	3.254	0.418	7.781	0.000
*R*-squared value: 0.130					*R*-squared value: 0.184				
Adjusted *R*-squared value: 0.120					Adjusted *R*-squared value: 0.174				
*F*-statistics vs. constant model: 12.4, *p* < 0.001					*F*-statistics vs. constant model: 18.7, *p* < 0.001				

*X* is the natural logarithm of age plus one. The coefficients of the terms in the quadratic fit to the transformed data are notated under B. SE stands for standard error. The degrees of freedom for the F-statistics were (2, 166).

**Table 2:** Summary statistics of two-sample *t*-tests examining sex differences in the residuals of fits shown in Figure 2.

Measure	|*t*(167)|	*p*
Mean, initiation speed	0.015	0.99
SD, initiation speed	0.150	0.88
Mean, arrival speed	0.652	0.52
SD, arrival speed	0.592	0.56

## Discussion

We quantified visuomotor reaction speed performance on a two-dimensional saccade task in a cross-sectional sample of 169 healthy individuals aged 8–82 years. Inter-individual variability was evident at any age in the saccade reaction metrics, against which measurement reliability was demonstrated. A practice effect was not observed. However, there was a small unexplainable slowing of arrival speed on the second testing. Habituation to the simple stimulus or related decrease in motivation may be taken into consideration in future test designs.

The reliable inter-individual variations in normal saccade performance may represent an attribute that can contribute to multidimensional characterization of individual cognitive profiles when combined with other assessments that also demonstrate test–retest reliability and inter-individual variability but target different domains, such as ANAM, DANA, and the ImPACT (6–8, 37, 51). Further, despite the large inter-individual variability, we demonstrated the presence of U-shaped development-aging trend, as previously identified in one-dimensional tasks (24–26). Overall, the speed of saccade initiation was fastest and least variable in the 20s age range. The speed of bringing the gaze to the target, often utilizing multiple saccades, was fastest in the 20s, but least variable in the 30s. Since reactive saccade performance metrics may provide signatures of abnormal neurological or cognitive states (22, 30, 31, 36–40), further understanding of age-dependent variations in normal behavior is necessary.

### Limitations

We tested a cohort of civilians with a limited sample size for any given age. Because of the choice of the sample population, and as observed with other existing cognitive assessment tools (9), there may be a gap in establishing a link between our results with real-world task performance such as in a military or other specialized context. Also limiting the results’ ecological validity was that testing was conducted in a laboratory setting using specialized equipment. Further, about 10% of the collected data were invalidated due to quality issues. However, improved performance quality and availability of eye tracking technology using a tablet computer, smartphone, or head-mounted virtual reality system (52–54) may permit a real-time practical application of a reactive saccade test such as one we studied to assess cognitive readiness.

## Conclusion

We verified within-individual stability and across-age differences in reactive saccade performance. A practice effect was not associated with the task, making such tests well-suited for repeated administration. Variations in normal behavior may represent different cognitive attributes. These test results support cognitive surveillance for quickly assessing normal and impaired attention, detecting changes in attentional states within individuals over time, and informing immediate and long-term task assignment in military operations or return-to-play decisions in sports.

## Data Availability

The datasets presented in this article are not readily available because raw data were collected with specialized equipment and may contain information traceable to individuals. Requests to access the datasets should be directed to Jun Maruta, jun.maruta@mssm.edu.
